# Hereditary angioedema with C1 inhibitor deficiency: delay in diagnosis in Europe

**DOI:** 10.1186/1710-1492-9-29

**Published:** 2013-08-12

**Authors:** Andrea Zanichelli, Markus Magerl, Hilary Longhurst, Vincent Fabien, Marcus Maurer

**Affiliations:** 1 Department of Clinical Science “Luigi Sacco”, University of Milan, Milan, Italy; 2 Department of Dermatology and Allergy, Allergie-Centrum-Charité, Charité-Universitätsmedizin Berlin, Berlin, Germany; 3 Bart’s and The London Hospital, London, UK; 4 Shire Human Genetic Therapies Inc., Eysins, Switzerland

**Keywords:** Bradykinin, C1-inhibitor, Diagnosis, Hereditary angioedema, Icatibant

## Abstract

**Background:**

Hereditary angioedema (HAE) is a rare, debilitating, and potentially life-threatening disease characterized by recurrent edema attacks. Important advances in HAE treatment have been made, including the development of new therapies for treating or preventing attacks. Nevertheless, the disease is still frequently misdiagnosed and inappropriately treated, potentially exposing patients with laryngeal attacks to the risk of asphyxiation.

**Methods:**

The Icatibant Outcome Survey (IOS) is an international, observational study that documents the clinical outcome of HAE patients eligible for treatment with icatibant. Patient ages at first symptoms and at diagnosis were recorded at enrolment, and the delay between first symptoms and diagnosis was calculated.

**Results:**

The median [range] diagnostic delay in HAE type I and II patients across eight countries was 8.5 years [0–62.0]. The median delay in diagnosis was longer for HAE type II versus type I (21 versus 8 years, respectively), although this did not quite reach statistical significance.

**Conclusions:**

Although it can be difficult to differentiate HAE symptoms from those of more common angioedema sub-types (e.g. idiopathic or acquired angioedema), our results show that HAE type I and II patients have an unacceptable delay in diagnosis, even those with a family history of the disease. Raising physician awareness of this disabling and potentially fatal disease may lead to a more accurate diagnosis and timely treatment.

## Background

Hereditary angioedema (HAE), due to C1-inhibitor (C1-INH) deficiency, is a rare disease with an estimated frequency of 1:50 000 [1]. HAE is caused by mutations in the C1-INH gene that results in reduced levels (type I) or function (type II) of C1-INH protein [2]. The deficiency of C1-INH leads to increased activation of the contact system, which generates elevated levels of bradykinin (the mediator of the increased vascular permeability) and the resulting edema observed in HAE patients [3]. The clinical features of HAE include recurrent episodes of edema, which usually last for 2–5 days. The skin, gastrointestinal tract, and upper airway are most commonly affected. Delayed treatment of laryngeal swelling can result in death [1,4].

The rarity of HAE, the fact that its symptoms overlap with those of other forms of angioedema and, in cases of abdominal attack, can appear to be a surgical emergency, mean that it is frequently misdiagnosed. Consequently, HAE patients may experience considerable delays in diagnosis [5,6]. Previous nationwide surveys in Spain and Denmark have reported mean delays in diagnosis of 13.1 and 16.3 years, respectively [5,6]. In an international web-based survey, 313 patients with HAE reported visiting an average of 4.4 physicians over an average of 8.3 years before receiving an accurate HAE diagnosis [7]. Without an accurate diagnosis, HAE patients may not receive treatment that can effectively treat their attacks. Inappropriate treatment could result in higher morbidity and mortality, adverse events, and unnecessary surgical interventions [8,9].

Recently published studies and guidelines [10-18] have resulted in an increased awareness of HAE in the scientific community; however, HAE remains under-diagnosed. The Icatibant Outcome Survey (IOS) is an international, observational study that documents the clinical outcome of HAE patients eligible for treatment with icatibant. Here, we report the delay in diagnosis in adult patients with type I or II HAE.

## Material and methods

### Participants

IOS registry data from 171 HAE type I and II patients (female/male: 105/66; HAE type I/II: 158/13) from centers in Germany (N = 46), Spain (N = 45), Italy (N = 30), France (N = 18), the UK (N = 17), Denmark (N = 13), Israel (N = 1) and Sweden (N = 1) were analyzed.

HAE type I was diagnosed in symptomatic patients when both C1-INH concentration and function were below normal; HAE type II was diagnosed in symptomatic patients when C1-INH concentration was normal or above normal, and function was below normal. The normal ranges (according to local reference values) for C1-INH concentration and function were 15–50 mg/dL and 70–130%, respectively. Patients diagnosed prior to the onset of symptoms, i.e. on the basis of family history, were excluded from this analysis.

### Study design and setting

IOS is an international, multicenter, observational patient registry for patients treated with icatibant (Firazyr®). This analysis is based on data collected between July 2009 and June 2012. IOS is conducted in accordance with the Declaration of Helsinki, and the International Conference on Harmonization Good Clinical Practice Guidelines. After approval from local Ethics Committees and/or Health Authorities (where applicable) had been obtained by the center, all patients provided written informed consent.

Demographic and diagnostic information were collected from the patient at enrollment. Patient ages at first symptoms and at diagnosis were recorded, and the delay between first symptoms and diagnosis was calculated.

### Statistical analyses

In order to compare the age at diagnosis, the age at first symptoms, and the delay between first symptoms and diagnosis, the Wilcoxon–Mann–Whitney test was used. The one-way analysis of variance with multiple comparisons (‘least significant difference method’ from Fisher) was used for the comparison between countries (Sweden and Israel were excluded from this analysis due to low patient numbers). The level of statistical significance chosen was alpha = 0.05.

## Results

The median [range] delay in diagnosis in HAE type I and II patients (N = 152) was 8.5 years [0.0–62.0]. The median age at first symptoms (N = 153) was 12.0 years [0.2–77.0] and the corresponding median age at diagnosis (N = 170) was 24.3 years [0.0–77.3]. The difference observed in the delay in diagnosis between patients with HAE type I and patients with HAE type II was almost significant (8.0 [0.0–62.0] versus 21.0 years [0.0–42.0]; p = 0.051). However, no difference in delay in diagnosis was found between female and male patients, or between symptomatic patients with a positive and a negative family history (Table 1).

**Table 1:** Age at first symptoms and diagnosis, and delay in diagnosis by patient type

		**N**	**Median**	**Q1**	**Q3**	**Min**	**Max**	**Mean**	**Standard deviation**	**p-value**
Age at first symptoms (years)	Male	61	13.0	6.0	20.0	1.3	60.0	15.9	12.53	0.199
	Female	92	11.5	5.0	18.0	0.2	77.0	13.6	12.15	
	HAE type I	140	12.0	6.0	19.0	0.2	77.0	14.4	12.29	0.870
	HAE type II	13	13.0	6.0	18.0	2.0	50.0	15.3	13.12	
	FH+	106	13.0	6.0	19.0	0.2	50.0	13.7	9.42	0.513
	FH-	32	14.0	5.0	26.5	0.8	77.0	19.6	19.60	
	**Total**	**153**	**12.0**	**6.0**	**19.0**	**0.2**	**77.0**	**14.5**	**12.32**	
Age at diagnosis (years)	Male	66	25.1	17.9	33.0	9.1	68.3	27.6	14.06	0.844
	Female	104	23.3	15.8	38.3	0.0	77.3	28.1	17.37	
	HAE type I	157	22.1	16.1	36.0	0.0	77.3	27.3	15.87	0.080
	HAE type II	13	31.8	24.2	43.5	3.3	74.2	34.9	18.18	
	FH+	121	24.4	16.2	35.2	0.0	74.2	26.9	14.96	0.509
	FH-	33	22.3	17.1	45.0	3.8	77.3	30.5	19.10	
	**Total**	**170**	**24.3**	**16.9**	**36.2**	**0.0**	**77.3**	**27.9**	**16.13**	
Delay between first symptoms and diagnosis (years)	Male	60	8.5	3.0	17.0	0.0	60.0	11.2	10.97	0.771
	Female	92	8.5	1.0	24.0	0.0	62.0	13.9	14.88	
	HAE type I	139	8.0	2.0	20.0	0.0	62.0	12.2	13.35	0.051
	HAE type II	13	21.0	7.0	30.0	0.0	42.0	19.6	13.80	
	FH+	106	8.5	2.0	21.0	0.0	57.0	12.5	12.94	0.476
	FH-	31	6.0	1.0	20.0	0.0	62.0	10.8	13.38	
	**Total**	**152**	**8.5**	**2.0**	**21.0**	**0.0**	**62.0**	**12.9**	**13.5**	

FH+, positive family history; FH-, negative family history; Q1, lower quartile; Q3, upper quartile.

The diagnostic delay was markedly different between countries; ranging from 2.0 years [0.0–62.0] (Germany) to 15.0 years [0.0–57.0] (Italy) (Table 2). However, there were no trends or statistical significant differences in diagnostic delay between countries (p = 0.1583), even when analyzed by year of diagnosis (before 1990, 1990–2000, after 2000).

**Table 2:** Delay in diagnosis by country

	**N**	**Median (years)**	**Q1**	**Q3**	**Min**	**Max**	**Mean**	**Standard deviation**
Germany	41	2.0	0.0	9.0	0.0	62.0	9.0	13.91
Spain	40	13.0	3.5	21.5	0.0	60.0	15.4	13.74
Italy	30	15.0	4.0	21.0	0.0	57.0	15.7	14.14
France	15	7.0	4.0	24.0	2.0	38.0	14.3	13.47
Denmark	12	11.5	3.0	22.5	0.0	34.0	13.6	11.68
UK	12	5.5	1.0	16.5	0.0	20.0	8.0	7.79
**All**	**150**	**8.5**	**2.0**	**21.0**	**0.0**	**62.0**	**12.8**	**13.47**

Due to the low number of patients in Sweden (N = 1) and Israel (N = 1), these patients have been excluded from the analysis.

Nine (5.3%) of the 171 patients included in the IOS database were diagnosed with HAE type I or II based on their family history and prior to the onset of symptoms. These patients therefore have negative delay in diagnosis values and were excluded from the analyses above.

## Discussion

Our study demonstrates that, in a sizable population of HAE patients from eight countries, the median delay in diagnosis is 8.5 years [range 0.0–62.0]. Possible explanations for the difference in delay in diagnosis between countries (although not statistically significant; p = 0.1583) include awareness of HAE among physicians, patient selection bias during IOS enrolment, or demographic differences between national patient populations.

We found a considerable difference in delay in diagnosis between patients with HAE type I and II, although not significant (8.0 [0.0–62.0] versus 21.0 years [0.0–42.0]; p = 0.051). This difference might be due to the fact that patients are only tested for C1-INH levels, which are normal or elevated in HAE type II patients. To avoid further delay in diagnosis of type II patients, functional C1-INH analysis also needs to be performed.

Interestingly, patients with a positive HAE family history were not diagnosed earlier than those with a negative family history and, currently, only nine (5.3%) of the 171 patients enrolled in IOS were diagnosed prior to the onset of symptoms, i.e. on the basis of family history. This indicates that patients may not be adequately informed about the hereditary nature of their disease or, alternatively, may choose not to seek diagnosis. Undiagnosed patients are at a greatly increased risk of premature death from asphyxiation and of the social and economic consequences of untreated HAE [9,18]. Active screening of family members for C1-INH deficiency, access to effective, well-tolerated treatment options, and education of patients and their families with regards to the benefit of diagnosis and treatment are necessary to improve this unsatisfactory situation.

## Conclusion

Patients with HAE continue to have an unacceptable delay (median 8.5 years [0.0–62.0]) between the time they experience their first attack and are adequately diagnosed. This delay could be reduced by increasing awareness of HAE in the scientific community, as well as among patients. Since it might be difficult to differentiate HAE symptoms from those of more common angioedemas; when HAE is suspected, laboratory analysis for C1-INH levels *and* function can confirm the deficiency and guide physicians in making a correct diagnosis and initiating appropriate treatment.

## Abbreviations

C1-INH: C1 inhibitor; HAE: Hereditary angioedema; IOS: Icatibant outcome survey.

## Competing interests

Dr Zanichelli is an advisor for Shire Human Genetic Therapies (HGT) Inc. and has been an invited speaker for Shire HGT Inc., CSL Behring, and Swedish Orphan Biovitrum.

Dr Magerl has acted in a consultant/advisor capacity, has been an investigator in a company-sponsored scientific study, has received a travel grant for presenting at a scientific congress, and/or has lectured/spoken at company-sponsored meetings for CSL Behring, Shire HGT Inc., Swedish Orphan Biovitrum, and ViroPharma.

Dr Longhurst has received funding for research and staff support from CSL Behring, Shire HGT Inc., and Pharming; is a consultant for CSL Behring, Dyax, Shire HGT Inc., Swedish Orphan Biovitrum, and ViroPharma; and a speaker for CSL Behring, Shire HGT Inc., Swedish Orphan Biovitrum, and ViroPharma.

Dr Fabien is an employee of Shire HGT Inc.

Dr Maurer has received speaker/consultancy fees from Biocryst, Shire HGT Inc./Jerini AG, and ViroPharma.

## Authors’ contributions

All authors made equal contributions to the development of the publication. All authors read and approved the final manuscript.
